# Distribution and Geochemical Controls of Arsenic and Uranium in Groundwater-Derived Drinking Water in Bihar, India

**DOI:** 10.3390/ijerph17072500

**Published:** 2020-04-06

**Authors:** Laura A. Richards, Arun Kumar, Prabhat Shankar, Aman Gaurav, Ashok Ghosh, David A. Polya

**Affiliations:** 1 Department of Earth and Environmental Sciences and Williamson Research Centre for Molecular Environmental Science, The University of Manchester, Williamson Building, Oxford Road, Manchester M13 9PL, UK; 2 Mahavir Cancer Sansthan and Research Center, Phulwarisharif, Patna 801505, India

**Keywords:** arsenic, uranium, Bihar, India, groundwater quality, groundwater monitoring

## Abstract

Chronic exposure to groundwater containing elevated concentrations of geogenic contaminants such as arsenic (As) and uranium (U) can lead to detrimental health impacts. In this study, we have undertaken a groundwater survey of representative sites across all districts of the State of Bihar, in the Middle Gangetic Plain of north-eastern India. The aim is to characterize the inorganic major and trace element aqueous geochemistry in groundwater sources widely used for drinking in Bihar, with a particular focus on the spatial distribution and associated geochemical controls on groundwater As and U. Concentrations of As and U are highly heterogeneous across Bihar, exceeding (provisional) guideline values in ~16% and 7% of samples (*n* = 273), respectively. The strongly inverse correlation between As and U is consistent with the contrasting redox controls on As and U mobility. High As is associated with Fe, Mn, lower *Eh* and is depth-dependent; in contrast, high U is associated with HCO_3_^−^, NO_3_^−^ and higher *Eh*. The improved understanding of the distribution and geochemical controls on As and U in Bihar has important implications on remediation priorities and selection, and may contribute to informing further monitoring and/or representative characterization efforts in Bihar and elsewhere in India.

## 1. Introduction

Elevated concentrations of naturally occurring As and U in groundwater present a major environmental and public health challenge globally. Groundwaters within the major floodplains and deltas in South/Southeast (S/SE) Asia, as well as elsewhere across the world, can naturally contain dangerous concentrations of As [1,2,3,4,5] exceeding the World Health Organization (WHO) provisional guideline value of 0.13 µM (10 µg.L^−1^) [4]. In S/SE Asia, As impacts the health of millions of people and has been well documented in areas of Bangladesh [5,6,7,8,9,10,11,12,13,14,15], Cambodia [16,17,18,19,20,21,22,23,24,25,26,27,28,29], Vietnam [30,31,32,33,34,35,36,37,38,39,40,41], Pakistan [42,43,44,45] and more recently, Myanmar [46,47,48,49]. In India, groundwater As, particularly in the Ganga Basin, has been widely reported in West Bengal [50,51,52,53,54,55,56,57,58,59,60,61,62,63,64,65,66,67,68,69,70] (noting that a reduced As hazard as compared with previous estimates has recently been postulated in a Kolkata-based study [71]), Uttar Pradesh [72,73,74,75] and Bihar [76,77,78,79,80,81,82,83]. In Bihar specifically, As was first reported in 2003 in Bhojpur district [76]. In 2007, a largescale study of ~67,000 water sources reported elevated As in 11 districts of Bihar (Begusarai, Bhagalpur, Bhojpur, Buxar, Katihar, Khagaria, Munger, Patna, Samastipur, Saran and Vaishali, noting importantly that only sources within 10 km of the Ganga River were sampled) [77]. Most recently, it has been reported that 22 districts of Bihar are currently As-impacted, although no further details such as location, non-summarized data or the functional definition of “As-impacted” are included [78]. Further, wheat consumption has recently been identified as an emerging route of As exposure in Bihar [84]. Although thorough reviews of the status of As in the Gangetic Basin are published elsewhere [78,85], the estimated population at risk for As contamination in West Bengal is 26 million people, followed by Bihar (9 million), Uttar Pradesh (3 million), Assam (1.2 million), Manipur (1 million) and Jharkhand (0.4 million) [85].

Widespread in nature, U is a radionuclide which geogenically occurs in granite and other rock types. U in groundwater can occur due to natural mobilization processes under oxic conditions, and can also be associated with anthropogenic activities such as mining, coal and fuel combustion, emissions from the nuclear industry and the use of PO_4_-based fertilizers containing U [4]. As chronic exposure to U may lead to numerous adverse health impacts including bone toxicity and impaired renal function [86,87], WHO has set a provisional guideline value of 0.13 µM (30 µg.L^−1^), noting that Germany adopted a lower threshold of 0.04 µM (10 µg.L^−1^) in 2011 [88]. Elevated concentrations of U in groundwater have been identified across the globe, including in North America (e.g., Canada [89], the United States [90,91]), Europe (e.g., Finland [87], Sweden [92], Switzerland [93], the United Kingdom [94]) and Asia (e.g., Bangladesh [95], China [96,97], Korea [98], Mongolia [99], Pakistan [43] and in the lower Mekong delta [100]), noting this is a non-exclusive list. In the Indian context specifically, U has been reported in groundwater in the States of Andhra Pradesh [101], Bihar [102,103,104], Chhattisgarh [105], Haryana [106], Jammu & Kashmir [107], Jharkhand [108,109,110], Himachal Pradesh [111], Karnataka [112], Kerala [113], Punjab [114], Rajasthan [115], Uttar Pradesh [116] and West Bengal [70]. A partial review of the range of U concentrations encountered in various Indian states is included in [102,105]. In Bihar, to the authors’ knowledge, published U studies are sparse and have been limited to five southwestern districts of Auranagabad, Gaya, Jahenabad, Nalada and Nawada where U was reported in elevated concentrations in selected samples [102,104], ranging in concentration from 0.1 to ~240 µg.L^−1^ (~0.0004 to ~1 µM) [102], and in several blocks of Patna where U was below permissible limits [103]. The co-occurrence of groundwater As and U has recently been reported in the neighbouring state of Uttar Pradesh [117].

The mobility and fate of both As and U in groundwater are highly dependent on local redox conditions. For As, mobilization in shallow, reducing groundwaters typical to S/SE Asia is generally attributed to reductive dissolution of As-bearing Fe(III) minerals [1,118] via metal-reducing bacteria and labile organic matter [2,32,118,119,120,121]. Mechanistic questions regarding the nature of organic matter [6,15,23,25,26,61,121,122,123,124,125,126,127,128,129] and where in the subsurface As mobilization occurs [6,25,26,130,131,132,133,134] remain important in developing predictions of future changes in As hazard [6,24,26]. Additional competing processes such as adsorption/desorption [135,136,137] and co-precipitation [138] can also impact As mobility. Similarly to As, redox conditions are a major control on the mobilization and fate of U. The dominant species of U in aqueous solutions with oxic to suboxic conditions are the highly mobile U(VI) species [139], whereas the reduced species of U(IV) are relatively immobile [140]. Further processes, such as sorption/desorption, precipitation/dissolution and complexation with carbonates [88,141,142], humic acids [143] and other organics [144] can impact U mobility in groundwater.

Published groundwater studies particularly for Bihar remain relatively limited as compared with the neighbouring states in the Gangetic Basin of India. The lack of availability of systematic and representative (non-summarized) data in the public domain remains a major limitation to enable understanding of geochemical controls, prediction of future changes or developing effective monitoring and/or mitigation schemes. As such, the aim of this study is to systematically obtain, evaluate and interpret representative primary groundwater (inorganic) chemical quality data for Bihar, India, in order to (i) understand the distribution of geogenic groundwater contaminants, notably As and U, across Bihar; and to (ii) characterise the dominant geochemical conditions and processes likely to impact groundwater geochemistry in Bihar. This manuscript builds upon preliminary results initially presented by Richards et al. [145].

## 2. Materials and Methods

### 2.1. Field Area Description

**Figure 1:**
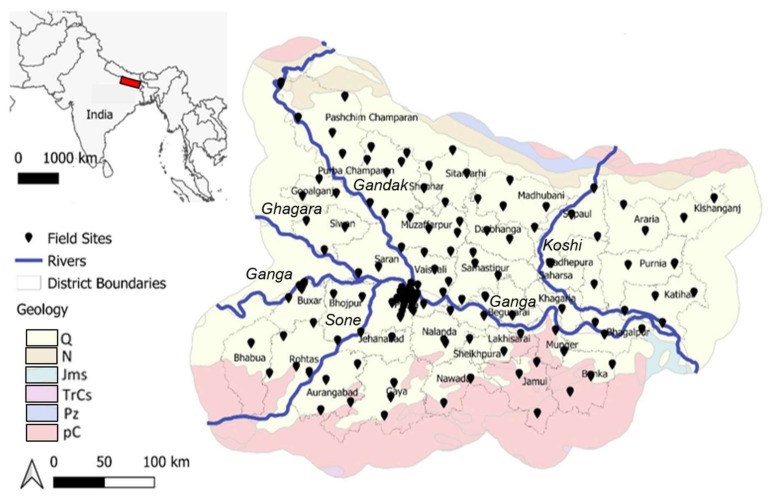
Groundwater sampling sites in Bihar, India [145] with major rivers labelled. Open-access layers are from Natural Earth [146], and geological layers from the US Department of Interior [147]. Geochronological categorization: Q = Quaternary; N = Neocene; Jms = Jurassic; TrCs = Triassic carboniferous; Pz = Paleozoic; pC = Pre-Cambrian. Area of detailed site map corresponds approximately to red box on regional map.

The study region is in the north-eastern state of Bihar, India, in the Middle Gangetic Plain and with a land area of ~94,000 km^2^ (Figure 1) [145]. Field sites were targeted in advance to encompass (i) representative sampling across all 38 districts of Bihar; (ii) minimized bias in sample selection; and (iii) logistic feasibility such as road access. The timing of samples was coordinated to avoid local election schedules for reasons of greater efficiency and safety. At least two (and ideally three) sampling depths were targeted at every site; in most cases, two depths were identified with local advice and usually were found easily within ~500 m of each target location. Depths were selected to capture the typical range of depths of groundwater wells used for drinking water in a particular area, and ranged from ~5 to 180 m depending on site. Higher-resolution sampling was undertaken in Patna district (*n* = 62; see Section 3.2 for detailed discussion) due to ongoing research in this area. With the total number of samples (*n* = 273) and an average of two depths collected per site, the approximate sampling density was two sites (of varying depths) per ~680 km^2^. Bihar is mostly dominated by recent Quaternary deposits and, in the south, much older pre-Cambrian features; further hydrogeological characterization of Bihar is reported elsewhere [81,82].

### 2.2. Water Sample Collection

Water sampling (*n* = 273) was conducted predominately in early and mid-2019 in the pre-monsoon season in Bihar, with the inclusion of a few additional samples collected in November 2018 (*n* = 3) and December 2019 (*n* = 4). Groundwater (from a depth range of approximately 5 to 180 m) was collected from existing private and government wells, either connected to a manual hand pump or on occasion an electrical submersible pump depending on the site. All sampled wells were in regular use and were pumped for at least ~1–2 minutes prior to collecting samples.

The subset of four samples from December 2019 were non-random samples collected from a known high-As area in Samastipur, an area of ongoing research by co-authors at Mahavir Cancer Sansthan for the purposes of external data verification; the results related to these samples are included for As only.

Subsamples of groundwater for cation, anion and dissolved organic carbon (DOC) analyses were immediately filtered (0.45 µm cellulose/polypropylene syringe filters, Minisart RC, Fisher Scientific, UK) upon collection, using similar methods as previously described [27]. Due to transport restrictions regarding nitric acid, subsamples for cation and trace metal(loid) analysis were acidified upon arrival at University of Manchester within the Manchester Analytical Geochemistry Unit (MAGU) laboratories using 2% trace analysis-grade nitric acid distilled in-house. All samples were stored in bottles which had been prepared in advance by acid wash (10% nitric acid wash) followed by thorough rinsing with deionized water (MilliQ grade) and furnacing at 400 °C to remove trace contamination.

### 2.3. Aqueous Analytical Measurements

Measurements on aqueous samples were conducted in both field and laboratory settings [27]. Field measurements included pH, oxidation–reduction potential (*Eh*), temperature and electrical conductivity, collected *in-situ* using Hanna handheld meters. Cations were analysed using inductively coupled plasma atomic emission spectrometer (ICP-AES, Perkin-Elmer Optima 5300 dual view; for analysis of Ca, Mg, Na, K, Si, Fe, Mn and P) and/or inductively coupled plasma mass spectrometry (ICP-MS, Agilent 7500cx; for analysis of As, U, Cu, Zn and Pb), both located within MAGU at The University of Manchester [27]. Anions were analysed using ion chromatography (IC; Dionex ICS5000 Dual Channel Ion Chromatograph; for analysis of F^−^, Cl^−^, NO_3_^−^, NO_2_^−^, SO_4_^2−^ and Br^−^) at MAGU. The method detection limit (also shown on Table 1) was 0.01 mg.L^−1^ for ICP-AES (corresponding to 0.0002 mM for Ca, Fe and Mn; 0.0003 mM for K and P; and 0.0004 mM for Mg, Na and Si) and 1 µg.L^−1^ for ICP-MS (corresponding to 0.01 µM for As; 0.02 µM for Cu and Zn; 0.004 µM for U; and 0.005 µM for Pb). For IC, the method detection limit was 0.1 mg.L^−1^ for Br^−^ and NO_2_^−^ (0.001 and 0.002 mM, respectively), 0.05 mg.L^−1^ for F^−^ (0.003 mM) and 0.2 mg.L^−1^ for Cl^−^, SO_4_^2−^ and NO_3_^−^ (0.006, 0.002 and 0.003 mM, respectively). Alkalinity was estimated by charge balance (e.g., the charge balance as calculated with measured concentrations of major cations, and measured concentrations of major anions including estimated alkalinity, was assumed to be zero); this parameter was not determined analytically during this study.

### 2.4. Quality Assurance and Quality Control

The quality assurance/quality control (QA/QC) measures undertaken during sampling and analyses were similar to as described in our previous work [27,148,149]. Method comparisons were undertaken to compare the impact of the simplified sampling strategy employed here (e.g., no acidification at the time of sampling) via comparison of in-lab filtration and acidification. Certified reference materials (CRMs) included SPS-SW1 (LGC Standards, Middlesex, UK), SRM 1640a (National Institute of Standards and Technology, USA), TM-25.5 (National Water Research Institute, Environment Canada) and CA011C (European Reference Material, LGC Standards, Middlesex, UK) for ICP-MS and ICP-AES, and LGC6020 (LGC Standards, Middlesex, UK) for IC. Duplicate analysis was conducted on 15% of the samples, and seven-point calibrations were repeated every 10 samples. Inverse variance weighted first order linear models were used for ICP-AES and ICP-MS calibration outputs [148,149].

### 2.5. Statistical Analysis and GIS

All statistical analysis was conducted using OriginPro 2017 and Microsoft Excel, and regression statistics are reported at 95% confidence as “*t(degrees of freedom) = t value; p = p value*”. Analyses of water samples with concentrations below method detection limits (as listed on Table 1) were treated as (i) zero in tabulated statistical calculations for quartiles, median and mean and (ii) as the method detection limit for correlation/regression calculations. In some Figures, including box plots, samples with measured concentrations below detection limit are included at the maximum feasible concentration (e.g., the method detection limit) to avoid bias or misinterpretation of data introduced by non-inclusion of below-detection samples; see figure captions for details. Maps were created using open-source QGIS (Version 3.10 A Coruña; https://qgis.org/en/site/), with open-access layers from Natural Earth [146] and geological layers from the US Department of Interior [147].

## 3. Results

### 3.1. Data Quality

The mean calculated bias for ICP-MS analytes across analytical runs reported here (March 2019, April 2019, July 2019 and January 2020), based on CRM SPS-SW1, is as follows: –1% for Cu, 3% for Zn, 0% for As and 4% for Pb. For U, bias is –5% at 25.4 µg.L^−1^ (CRM 1640a, only available for the March 2019 and January 2020 analytical runs) and 37% at 0.5 µg.L^−1^ (CRM SPS-SW1, noting the certified value is below the lowest calibration standard used of 1 µg.L^−1^). The July 2019 ICP-MS analytical run tended towards slightly higher analytical bias than the other analytical dates. For ICP-AES analytical runs in March, April and July 2019, the mean calculated bias on the basis of CRM SPS-SW1 is 0% for Fe, –3% for Mn, –7% for P, –11% for Ca, –12% for Mg, –20% for Na and –30% for K. For IC analytical runs in March and August 2019, mean calculated bias on the basis of CRM LGC6020 is –6% for Cl^−^, –8% for F^−^, –1% for NO_3_^−^ and –3% for SO_4_^2−^. No corrections were made on the basis of analytical bias, particularly due to the reasonable agreement observed for the parameters of primary interest. Methodological comparisons of filtration only in the field versus acidification (for a minimum of 48 h) followed by filtration in the laboratory showed reasonable agreement. Duplicate analyses were typically within ~10%.

### 3.2. Dominant Groundwater Geochemical Conditions

**Figure 2:**
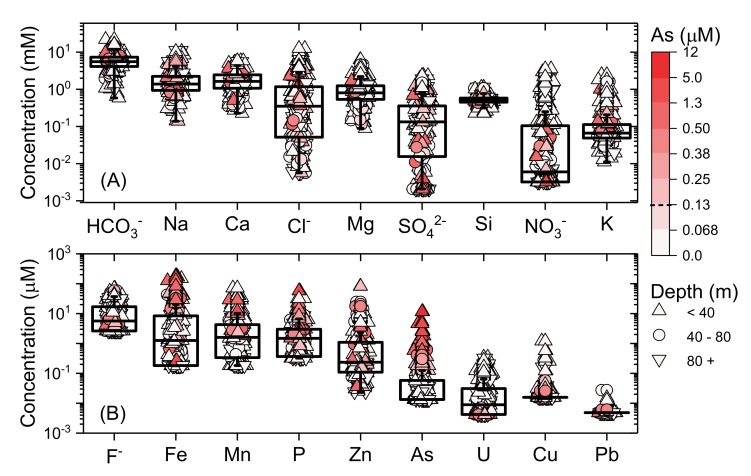
Box and Whisker diagram for (**A**) majors and (**B**) traces across Bihar. Boxes represent the 25%–75% distribution (central line within box represents the median), and lines indicating the 5% and 95% intervals. Colour scale indicates As concentrations, and symbol shape indicates reported depth. Samples with measured concentrations below detection for a particular analyte were calculated based on maximum concentration at method detection limit for inclusion in box plots; where median or lower whiskers are not clearly seen there are high numbers of samples below detection.

The major and trace elemental composition of Bihar groundwater is summarized in Figure 2. The bulk cationic charge is dominated by Na, Ca and Mg (contributing a max of 78%, 73% and 58%, respectively), followed by K, whereas HCO_3_^−^ and Cl^−^ dominate the bulk anionic charge (contributing a max of 99% and 63%, respectively), followed by SO_4_^2−^ and NO_3_^−^. Traces, in broad order of decreasing concentrations, include F^−^, Fe, Mn, P, Zn, As, U, Cu and Pb.

**Figure 3:**
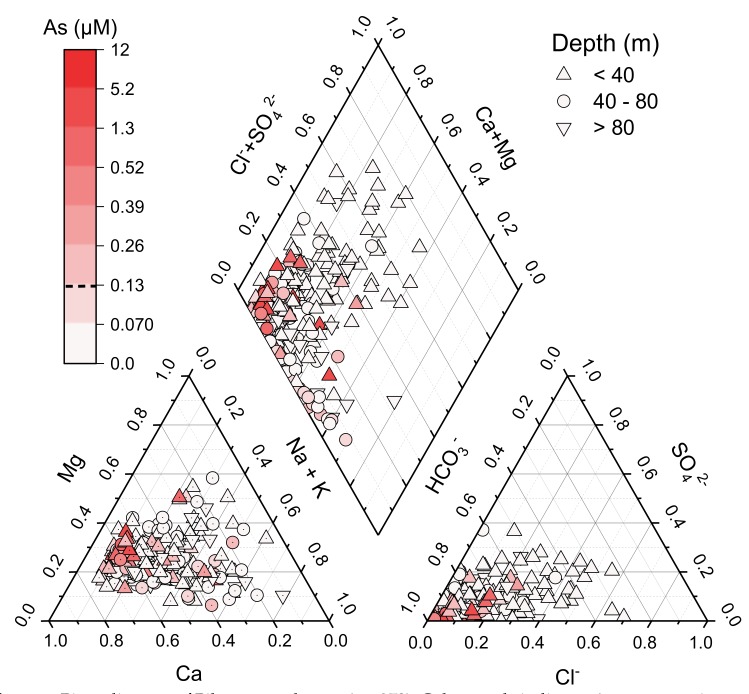
Piper diagram of Bihar groundwater (*n* = 273); Colour scale indicates As concentrations, and symbol shape indicates reported depth.

A Piper diagram (Figure 3) indicates the majority of the groundwater is of the Ca-HCO_3_^−^ type, with some samples instead Na-HCO_3_^−^ type. The dominance of Ca-HCO_3_^−^ water is typical of shallow groundwater, and the samples which instead reflect Na-HCO_3_^−^ type water suggest ion exchange reactions have influenced the groundwater composition. Most groundwater samples were circum-neutral (pH ranging from 5.7 to 8.3; median = 7.2), with a wide range of *Eh* (ranging from ~ 30 to 420 mV; median = 200 mV), electrical conductivity (EC; ranging from 0.03 to 3.1 mS.cm^−1^; median = 0.65 mS.cm^−1^) and relatively warm groundwater temperatures (~22 to 36 °C; median: 27 °C). Concentrations of groundwater As range from <0.01 to 11.6 µM (~ <1 to ~870 µg.L^−1^), with ~16% of samples exceeding the WHO provisional guideline of 0.13 µM (10 µg.L^−1^) [4], and ~4% exceeding 0.67 µM (50 µg.L^−1^). Groundwater U concentrations range from 0.0 to 0.35 µM (~<1 to ~80 µg.L^−1^), with ~7% of samples exceeding the WHO provisional guideline of 0.13 µM (30 µg.L^−1^). Neither As nor U is normally distributed at the 0.05 level according to the Shapiro–Wilk normality test. Full summary statistics are provided in Table 1.

**Figure 4:**
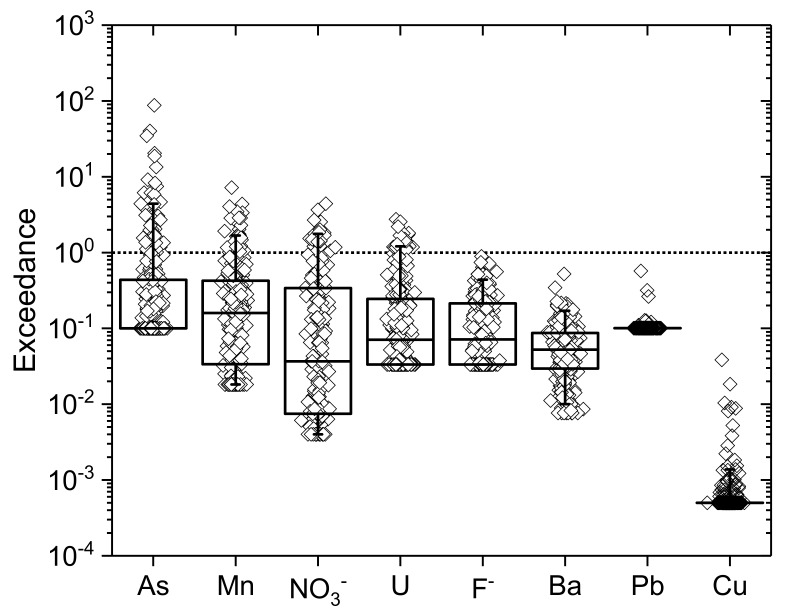
Exceedance plot for measured concentrations against WHO guideline values for As (0.13 µM; 10 µg.L^−1^; provisional), Mn (0.01 mM; 0.4 mg.L^−1^; former/discontinued), NO_3_^−^ (0.806 mM; 50 mg.L^−1^), U (0.13 µM; 30 µg.L^−1^; provisional), F^−^ (0.079 mM; 1.5 mg.L^−1^), Ba (0.0095 mM; 1.3 mg.L^−1^), Pb (0.048 µM; 0.01 mg.L^−1^; provisional) and Cu (0.032 mM; 2 mg.L^−1^). Exceedance was calculated by dividing measured concentrations by the relevant WHO guideline. Exceedance >1 indicates the number of samples above guideline values for each parameter (samples with measured concentrations below detection for a particular analyte were calculated based on maximum concentration at method detection limit for inclusion in exceedance plots). Median is line within box; boxes indicate the 25–75 percentile distribution, whiskers indicate the 5 and 95 percentile distribution. Where median or lower whiskers are not clearly seen there are high numbers of samples below detection.

An exceedance plot (Figure 4) with relation to WHO guideline values indicates that concentrations of As and U exceed guideline values in ~16% and ~7%, respectively, of measured samples. Concentrations of Mn exceed the previous WHO guideline in ~15% of samples. Although this guideline was discontinued in 2011 [150], high Mn groundwater can still be problematic, in particular due to concerns about risks associated with impairment of intellectual function [151] and/or for palpability and aesthetic reasons, and Mn reduction is a common remediation objective. NO_3_^−^ exceeds guideline values in ~8% of samples. Concentrations of F^−^, Ba, Pb and Cu are never in exceedance of guidelines for this sample set, however F^−^ in particular is very near guideline values (maximum concentration 0.07 mM; guideline value 0.08 mM) in a number of samples.

**Figure 5:**
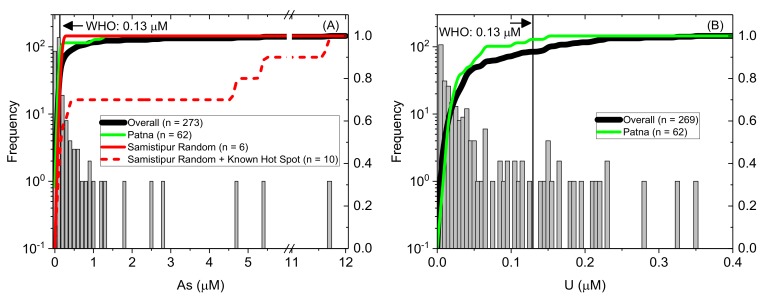
Histogram and cumulative frequency distribution curves for (**A**) As and (**B**) U in Bihar, India with WHO provisional guidelines [4] for As (0.13 µM; 10 µg.L^−1^) and U (0.13 µM; 30 µg.L^−1^) shown in solid lines. Histograms are shown for the overall dataset. The cumulative frequencies are included for the overall dataset (bold black line; *n* = 273 for As and *n* = 269 for U) and the Patna district subset (neon green line; *n* = 62). On (A) two lines for Samastipur are shown indicating one containing random sampling only (*n* = 6) and another with the inclusion of both random and samples from a known As “hot spot” (*n* = 10).

Given the particular focus on As and U in this manuscript, and the higher proportion of guideline exceedance for these parameters, histograms and cumulative frequency curves, including with comparison to WHO guideline values, are shown in Figure 5. In particular, we wish to highlight the higher sampling density undertaken in Patna district (*n* = 62) as compared with the overall dataset, in order to assess any potential disproportionate bias this may contribute. A comparison of cumulative frequency distribution curves indicates that although the Patna distribution is very similar to the overall Bihar dataset, Patna groundwater trends towards slightly lower concentrations of both As and U as compared with the overall dataset (shifting the cumulative distribution slightly left for As and slightly up for U). This is not expected to have a major influence on the results presented. On the other hand, in some cases sampling was biased to include locations where As had already been reported by co-authors—for example, in a Samastipur district known As “hot spot” location. This “hot spot” has markedly different characteristics than the random sampling would indicate (e.g., the cumulative distribution indicates much higher As than observed in the overall dataset). This has two very important implications on the interpretation presented here: (i) random sampling may miss areas of localized very high concentrations of groundwater contaminants; and (ii) in contrast, only sampling from known “hot spot” areas leads to a significant over-estimation of the concentrations encountered across a wider area. While we endeavoured to undertake representative sampling across Bihar, in some cases (e.g., in Samastipur and Buxar districts), our sampling included some sites of expected high As, which thus may bias some of the results reported for those districts. With these exceptions, sampling was conducted in order to minimize any sampling bias as much as possible. While we have endeavoured to undertake representative sampling across Bihar and to report any possible sampling bias with transparency, it is important to note that overall sampling density in this study remained relatively low. Thus, these results can not necessarily be considered comprehensive and in particular may not capture localized influences in particular locations which were not sampled. District-level variations are discussed in detail in Section 3.4.

### 3.3. Arsenic and Uranium Distribution in Bihar

**Figure 6:**
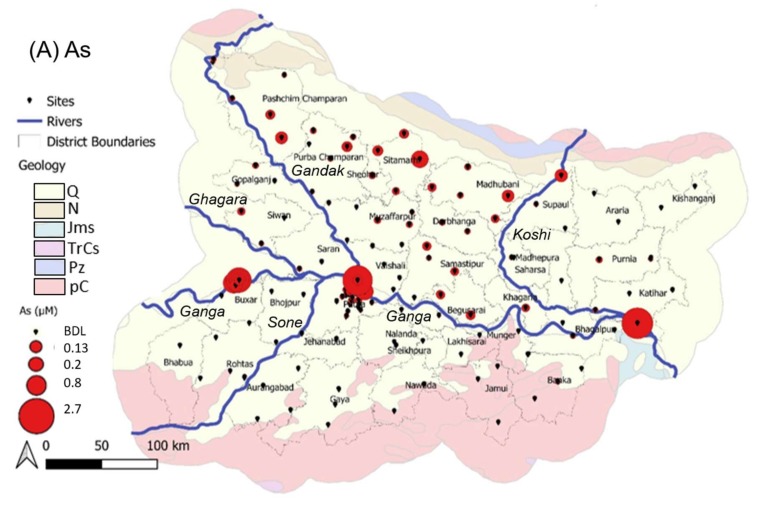

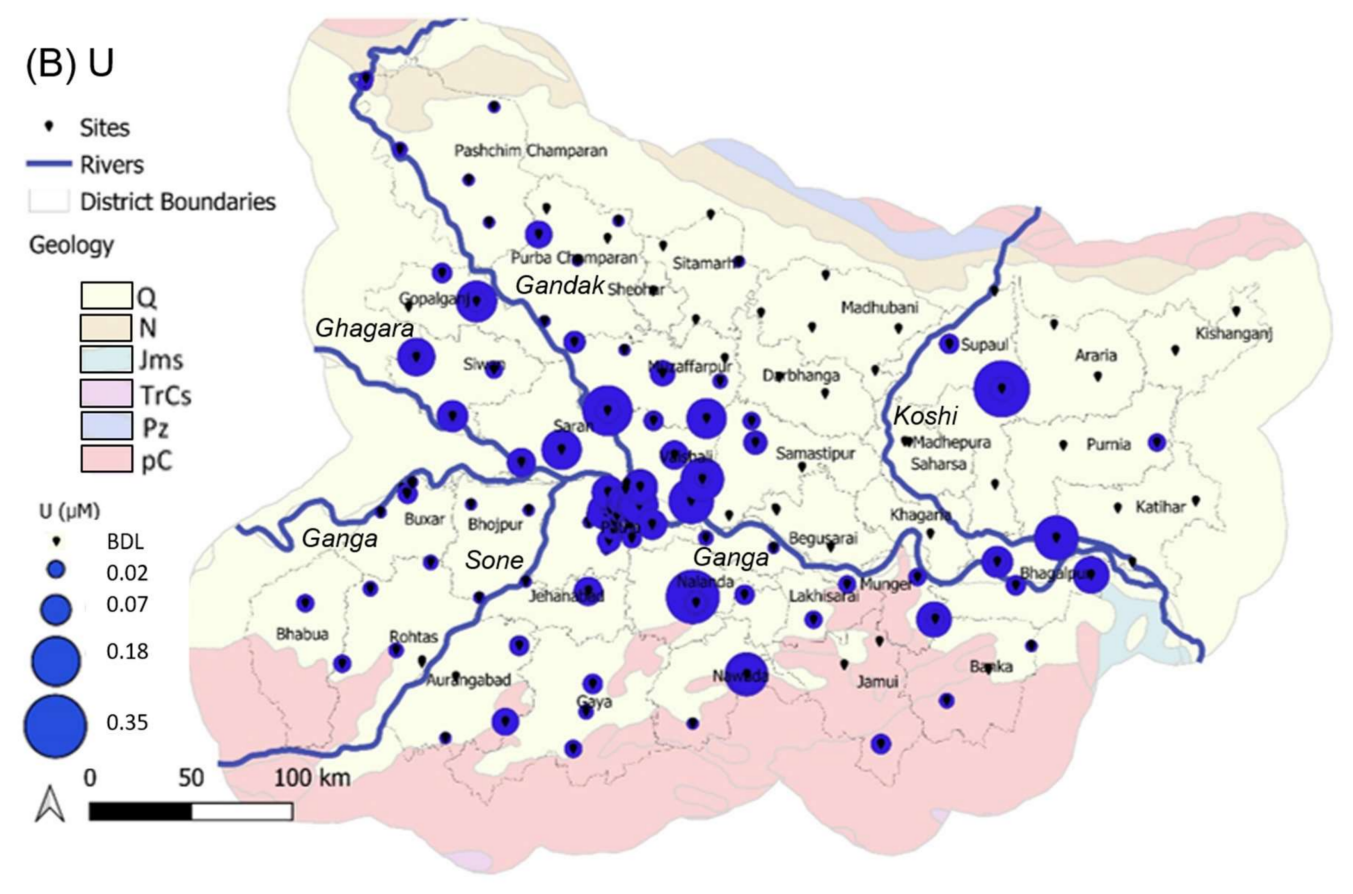
Spatial distribution of (**A**) As and (**B**) U in Bihar. Layers from Natural Earth and the US Department of Interior. Geochronological categorization: Q = Quaternary; N = Neocene; Jms = Jurassic; TrCs = Triassic carboniferous; Pz = Paleozoic; pC = Pre-Cambrian. BDL = below detection limit (0.01 µM for As and 0.004 µM for U).

The distribution of groundwater As and U across Bihar is presented (Figure 6). Concentrations of As (Figure 6A) tend to be higher in areas near and to the north of the Ganga River, particularly in the zone in between the Gandak and Koshi tributaries to the Ganga and near to the Nepal border. To our knowledge, detailed sampling has not been undertaken in the districts particularly in the far north of Bihar, where elevated As was found in some samples. The highest overall As concentration observed (11.6 µM) was in the non-randomized samples from a known “As hotspot” in Samastipur district which has been an area of ongoing investigations by co-authors (see discussion in Section 3.2; also noting that this point is not included on Figure 6A to avoid misinterpretation based on sampling bias). Following that, the highest concentrations were observed in Katihar (east Bihar, max 2.7 µM); Sabalpur (just north of the Ganga opposite Patna; max 2.5 µM) and in Buxar (max 1.8 µM). Both Patna (range <0.01 to 1.2 µM, *n* = 62) and Buxar (range < 0.01 to 1.7 µM, *n* = 15) districts had higher density of sampling, which may explain the wider range of As concentrations encountered in these districts. Areas south of the Ganga are consistently low in As.

The ~16% of samples here exceeding the WHO limit of 0.13 µM (10 µg.L^−1^) [4] is lower than other reports that ~33% of wells in Bihar (n ~ 20,000) exceed the same guideline [85]. In part, the higher sampling density in Patna district may contribute to this difference, although not to a major extent (see above discussion). The ~4% of samples here exceeding the previous Indian standard of 0.65 µM (50 µg.L^−1^) is also lower than that of an earlier study indicating that ~11% of samples (n ~ 67,000, all within 10 km of the Ganga River) exceeded 0.65 µM [77]. Differences could be due to sample size, sampling bias, methodology differences or changing geochemical conditions. Further, this highlights that As concentrations are highly heterogeneous, and suggests that the description of a particular district as simply “As-impacted” may not be particularly meaningful for informing public health risks. Geostatistical modelling of the distribution of groundwater As in Bihar is the subject of ongoing work by co-authors.

**Figure 7:**
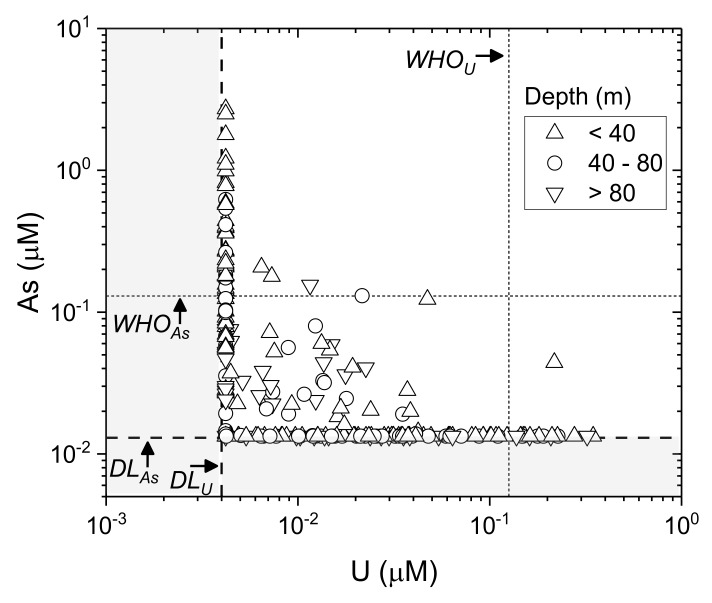
As versus U on a log-scale across the entire field area with WHO provisional guidelines [4] for As (0.13 µM; 10 µg.L^−1^) and U (0.13 µM; 30 µg.L^−1^) in dotted lines (WHO). Samples with measured concentrations below detection for a particular analyte are shown as maximum concentration at method detection limit (DL, shown in dashed lines). Grey boxes emphasize concentrations below method detection limits.

In contrast to As, U concentrations (Figure 6B) are elevated mostly in a NW–SE band along and to the east of the Gandak River and running south of the Ganga River towards Jharkhand, particularly in Gopalganj, Siwan, Saran, Patna, Nalanda and Nawada districts. There are also high-U samples in Supaul district (max 0.35 µM, the highest found in this study), which appear to be more isolated. Elevated U to the south of the Ganga, and especially in the southwestern districts of Aurangabad, Gaya, Jahenabad, Nalanda and Nawada, is consistent with previous work in Bihar [102]. In Patna, our results show that U varies significantly and is elevated in some parts of Patna, up to a max of 0.16 µM (38 µg.L^−1^); previously U has been reported to be well below permissible limits in several blocks of Patna [103]. In Singhbhum district (which was previously part of Bihar and now in Jharkhand State), located just south of Bihar, U mineralization is associated with Cu, Ni and sulphides in the Singhbhum shear zone, especially the U-enriched central zone between Jaduguda–Bhatin–Nimdih and Narwapahar–Garadih–Turamdih [152]. Elevated U concentrations have also been found in paleo-interfluvial groundwaters in the Bengal Basin [70]. Similar geological controls may impact the U trends observed here. Consistent with the spatial variability and the differences in redox controls on mobility, groundwater As and U concentrations are strongly inversely correlated (Figure 7; *t(267) = −2.4; p < 0.05*).

### 3.4. Distric-Specific Evaluation

**Figure 8:**
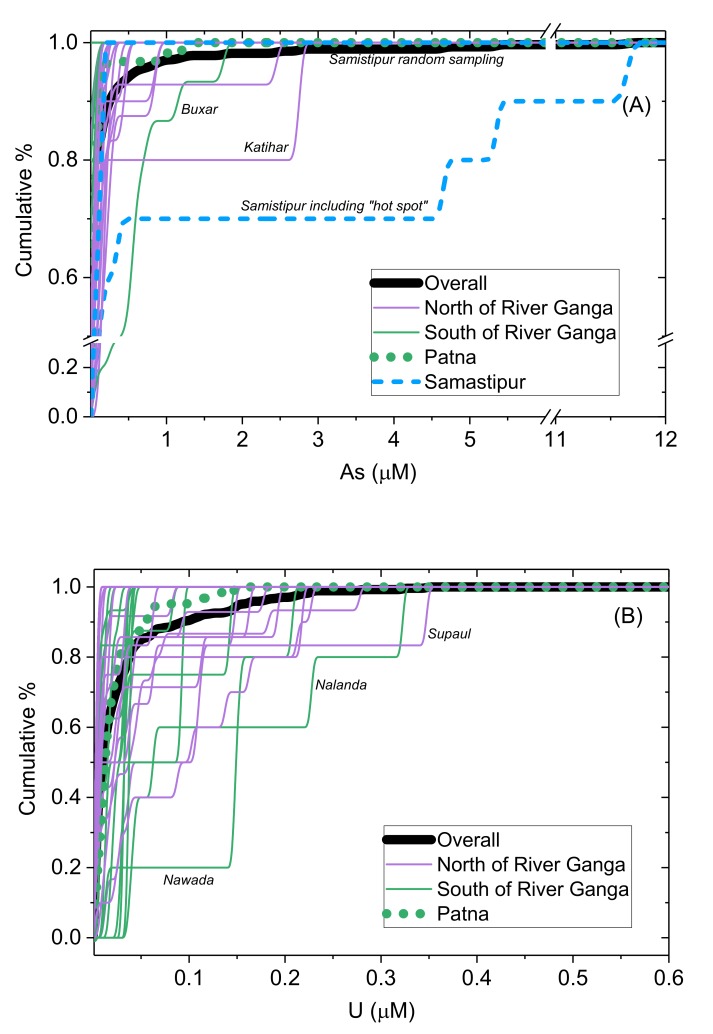
Cumulative distribution curves at a district level for (**A**) As and (**B**) U. Overall distribution is shown in bold black line; Patna in green dotted line. On (**A**), two lines for Samastipur (dashed blue line) are shown, indicating one containing random sampling only and another with the inclusion of samples from a known As “hot spot” (see discussion in Section 3.2). Colours indicate if districts are located north (purple) or south (green) of the River Ganga.

**Figure 9:**
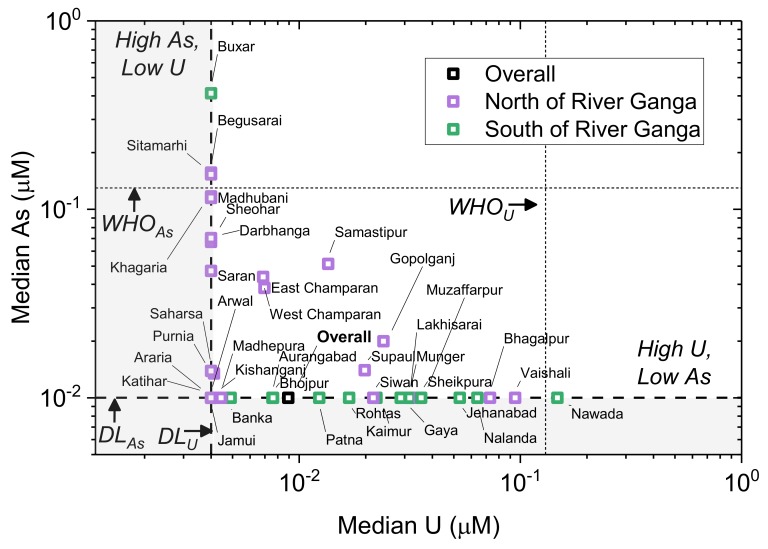
District-level median As versus median U in Bihar with district names labelled and geographic location (e.g., north versus south of Ganga River) indicated in colour. WHO provisional guidelines [4] for both As and U are shown in dotted lines (WHO); method detection limits (DL) for As and U are shown in dashed lines. Grey boxes indicate median concentrations below detection; districts with median concentrations of non-detection are presented at the value of the method detection limit to represent maximum (e.g., most conservative) concentration estimates.

District-level cumulative frequency distribution curves are shown for As and U (Figure 8). This helps to indicate the comparison at district level and is further supported by a summary of district-level As and U data (Table 2). This shows that the districts where As exceeded WHO provisional guidelines in at least one sample were Begusarai, Buxar, East Champaran, Katihar, Khagaria, Madhubani, Patna, Samastipur, Saran, Sitamarhi, Siwan, Supaul, Vaishali and West Champaran. Districts where U exceeded WHO provisional guidelines in at least one sample were Bhagalpur, Gopalganj, Katihar, Munger, Muzaffarpur, Nalanda, Nawada, Patna, Saran, Siwan, Supaul and Vaishali. There is a very strong geographical control, with districts south of the Ganga typically showing higher (district median) U and lower (district median) As than districts which are north of the Ganga (Figure 9).

### 3.5. Geochemical Controls on Arsenic Mobility

**Figure 10:**
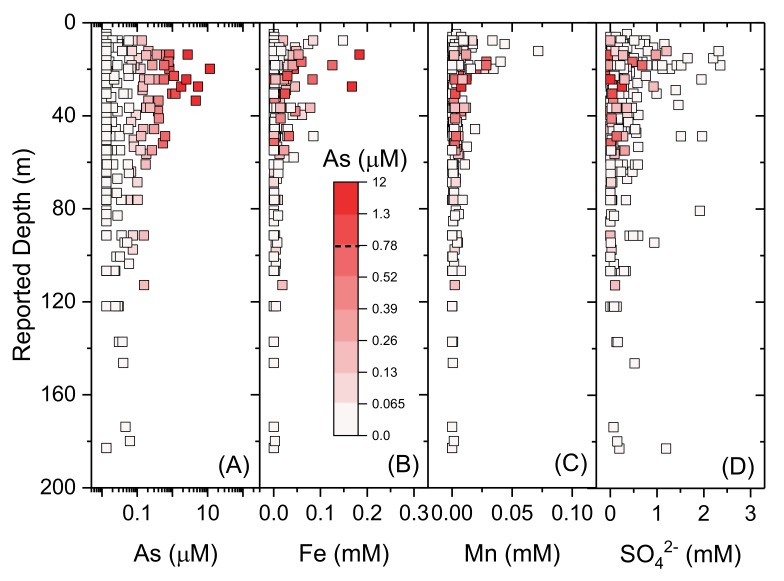
Depth profiles of (**A**) As (log scale), (**B**) Fe, (**C**) Mn and (**D**) SO_4_^2−^; colour scale indicates As concentrations with WHO provisional guideline [4] of 0.13 µM (10 µg.L^−1^). Samples with measured concentrations below detection for a particular analyte are shown as maximum concentration at method detection limit.

Concentrations of groundwater As show a broad dependence on depth (Figure 10A) (*t(269) = −1.5; p = 0.12*), which is consistent with previous studies, including in Bangladesh [6], Bihar [81,82] and beneath paleo-channels in West Bengal [69]. The highest As is most frequently found in the shallowest depths (<40 m), however importantly noting that elevated As is still found in some cases at depths of >100 m. Further, the same depth of groundwater supply can contain As concentrations varying by >2 orders of magnitude, highlighting the insufficiency of the anecdotal perception that depth is a suitable proxy for water quality. Depth profiles of Fe (Figure 10B), Mn (Figure 10C) and SO_4_^2−^ (Figure 10D) show similar depth dependence, with the highest concentrations of each of these parameters observed in shallow groundwater. Concentrations of As are strongly positively correlated with Fe (*t(267) = 10.9; p < 0.05*) and Mn (*t(267) = 2.4; p < 0.05*), and inversely correlated strongly with *Eh* (*t(236) = −2.9; p < 0.05*) as well as broadly with SO_4_^2−^ (*t(267) = −1.8; p = 0.07*). Elevated As associated with Fe and Mn in relatively low *Eh* and low SO_4_^2-^ groundwaters is highly consistent with As mobilization via reductive dissolution of Fe- and/or Mn- (hydr)oxides [26,27,118,153]. Bihar is known to be underlain by a two-tier aquifer system within a depth of ~ 300 m, separated by a clay/sandy clay aquitard ranging in thickness ranging from ~15–30 m [81]. Our findings here are consistent with previous observations that shallow (e.g., <~50 m) groundwater is particularly susceptible to As contamination, whereas deeper, confined or semi-confined aquifers typically are lower in As [81].

### 3.6. Geochemical Controls on Uranium Mobility

**Figure 11:**
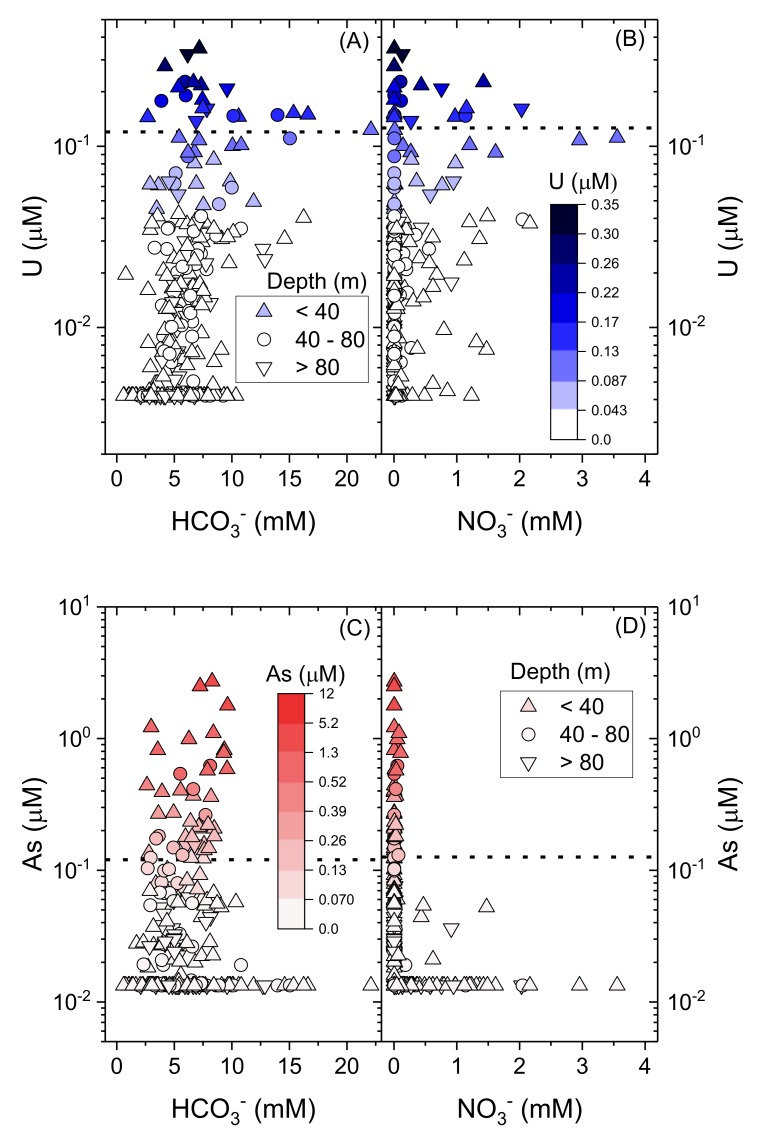
U (log-scale) versus (**A**) estimated HCO_3_^−^ and (**B**) NO_3_^−^ with the WHO provisional guideline [4] of 0.13 µM (30 µg.L^−1^) for U in dotted line. As (log-scale) versus (**C**) estimated HCO_3_^−^ and (**D**) NO_3_^−^ with the WHO provisional guideline [4] of 0.13 µM (10 µg.L^−1^) for As in dotted line. Symbol shape indicates reported depth; colour scale indicates U (blue) and As (red) concentrations. Samples with measured concentrations below detection for a particular analyte are shown as maximum concentration at method detection limit.

**Figure 12:**
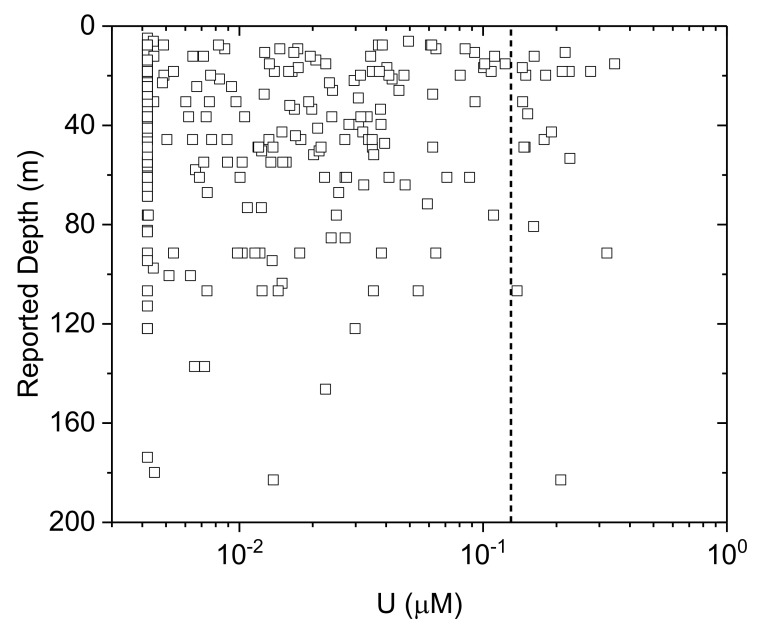
Depth versus U (log-scale) with the WHO provisional guideline [4] of 0.13 µM (30 µg.L^−1^) for U in dotted line. Samples with measured concentrations below detection are shown as maximum concentration at method detection limit.

The geochemical conditions favourable to support U release and/or mobility are strongly in contrast to those conditions in which As is present. U is instead associated with more oxidizing conditions, exhibiting a strong positive correlation with *Eh* (*t(232) = 3.5; p < 0.05)*). Further, U is strongly correlated both with HCO_3_^−^ (Figure 11A; *t(267) = 5.1; p < 0.05*) and with NO_3_^−^ (Figure 11B; *t(267) = 4.6; p < 0.05*). In contrast, for As, there is no statistically significant relationship with HCO_3_^−^ nor NO_3_^−^ (*p > 0.05* for both; Figure 11C,D). There is no apparent relationship between U and depth (*t(265) = -0.5; p = 0.6*) (Figure 12).

**Figure 13:**
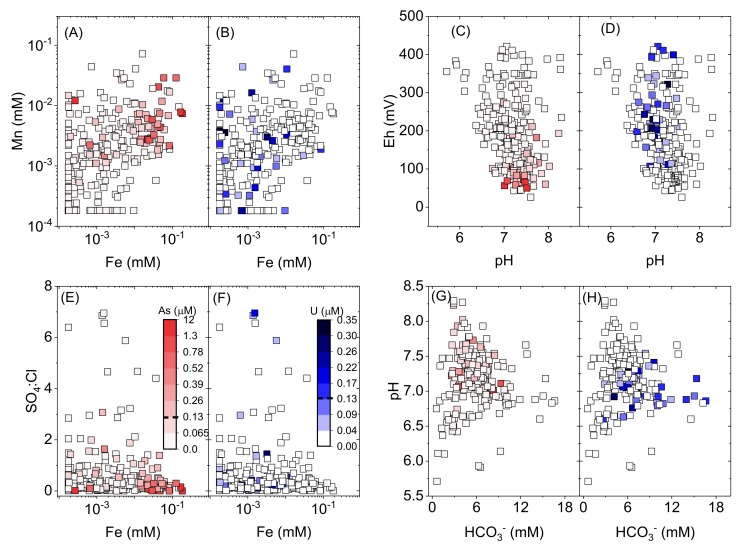
Bivariate plots for (**A**,**B**) Mn versus Fe; (**C**,**D**) *Eh* versus pH; (**E**,**F**) SO_4_^2−^:Cl^−^ (molar ratio) versus Fe; and (**G**,**H**) pH versus estimated HCO_3_^−^. Red colour scales (**A**,**C**,**E**,**G**) represent As concentrations, and blue colour scales (**B**,**D**,**F**,**H**) represent U concentrations. Samples with measured concentrations below detection for a particular analyte are shown as maximum concentration at method detection limit.

The contrasting As and U behaviours can be examined more closely by looking at various bivariate relationships (Figure 13). It is apparent that within the relationship between Mn and Fe (Figure 13A,B), high As is associated with high Mn and Fe (consistent with Figure 10), whereas the high U trends toward lower values of Fe in particular. Similar clustering of high-As groundwater is observed with high Fe and low SO_4_^2−^:Cl (molar ratio), whereas U is associated with higher SO_4_^2−^:Cl (Figure 13E,F). High As is correlated with relatively higher pH values, low *Eh* and lower HCO_3_^−^, whereas U trends towards lower pH and particularly higher *Eh* and HCO_3_^−^ (Figure 13C,D,G,H). A detailed investigation regarding the impact of surface-derived organic matter ingress, and associated changing redox conditions in the subsurface, on As mobilization in Patna, Bihar is the subject of ongoing work by co-authors.

## 4. Conclusions

In this study we characterize the inorganic major and trace element geochemistry in groundwater sources typically used for drinking across all districts of Bihar, India, with a particular focus on As, U and associated parameters. In Bihar, the dominant groundwater type is Ca-HCO_3_^−^ type, with some samples instead Na-HCO_3_^−^ type. Concentrations of As and U exceeded WHO (provisional) guideline values in ~16% and 7% of samples, respectively, as well as Mn (~15%) and NO_3_^−^ (~8%). Concentrations of F^−^, Ba, Pb and Cu were not observed to exceed guideline values in this sample set. Groundwater As and U were strongly inversely correlated, with higher As generally prevalent in reducing conditions and near and to the north of the Ganga river, and higher U typically in oxidizing conditions, particularly located in a NW–SE band running near and to the east of the Gandak River and to the south of the Ganga River. Concentrations of As were positively correlated with Fe and Mn, and inversely correlated with *Eh*, which is consistent with the mechanism of reductive dissolution of Fe- and/or Mn- (hydr)oxides. The strong depth-dependence of As is consistent with previous studies in Bihar and elsewhere in S/SE Asia. In contrast, elevated uranium was positively associated with *Eh*, HCO_3_^−^ and NO_3_^−^, with no apparent dependence on depth. Despite the relatively low sampling density, the spatial coverage across all districts in Bihar provides, for the first time, a comprehensive and consistent dataset for (inorganic) geochemical groundwater characterization in Bihar. These results have important implications for remediation priorities and the selection of appropriate remediation strategies, as well as for identifying areas where further representative sampling and/or monitoring may be beneficial.

## Figures and Tables

**Table 1:** Summary statistics for geochemistry of Bihar groundwater; MDL = method detection limit; Q = quartile; n/a = not applicable. Samples with measured concentrations below detection for a particular analyte are included as zero in summary statistic calculations.

Analyte	MDL	Min	Q1	Median	Q3	Max
pH (--)	n/a	5.7	7.0	7.2	7.5	8.3
*Eh* (mV)	n/a	30	110	200	240	420
Elec. Cond. (mS.cm^−1^)	n/a	0.03	0.5	0.7	0.9	3.1
Temp. (°C)	n/a	22.4	26.0	27.3	28.5	36.0
Na (mM)	0.0004	0.1	0.9	1.4	2.2	11
Ca (mM)	0.0002	0.2	1.1	1.6	2.5	5.9
Mg (mM)	0.0004	0.09	0.5	0.8	1.2	6.2
Si (mM)	0.0004	0.2	0.5	0.5	0.6	1.2
K (mM)	0.0003	0.01	0.05	0.07	0.11	2.5
HCO_3_^−^ (mM)	n/a	0.6	4.1	5.6	7.3	22
Cl^−^ (mM)	0.006	<0.006	0.1	0.4	1.2	12.6
SO_4_^2−^ (mM)	0.002	<0.002	0.02	0.13	0.4	2.4
NO_3_^−^ (mM)	0.003	<0.003	<0.003	0.006	0.1	3.4
F^−^ (mM)	0.003	<0.003	<0.003	0.006	0.02	0.07
P (mM)	0.0003	<0.0003	0.0004	0.002	0.003	0.06
Fe (mM)	0.0002	<0.0002	0.0002	0.001	0.01	0.2
Mn (mM)	0.0002	<0.0002	0.0003	0.002	0.004	0.07
Zn (µM)	0.02	0.02	0.11	0.24	1.1	82
As (µM)	0.01	<0.01	<0.01	<0.01	0.06	11.6
U (µM)	0.004	<0.004	<0.004	0.009	0.03	0.4
Cu (µM)	0.02	<0.02	<0.02	<0.02	<0.02	1.2
Pb (µM)	0.005	<0.005	<0.005	<0.005	<0.005	0.03

**Table 2:** Summary statistics for geochemistry of Bihar groundwater. District-level and overall summary statistics for As and U show minimum, median, mean and maximum concentrations (C_min_, C_median_, C_mean_, C_max_, respectively) and percentage of samples exceeding WHO provisional guideline values (Ex). WHO provisional guidelines [4] are 0.13 µM (10 µg.L^−1^) for As and 0.13 µM (30 µg.L^−1^) for U. Bold/underlined font indicate where C_median_, C_mean_ or C_max_ exceeds WHO guidelines. Samples with measured concentrations below detection for a particular analyte are included as zero in summary statistic calculations.

District	n	As					U				
		C_min_ (µM)	C_median_ (µM)	C_mean_ (µM)	C_max_ (µM)	Ex (%)	C_min_ (µM)	C_median_ (µM)	C_mean_ (µM)	C_max_ (µM)	Ex (%)
Overall	273	<0.01	<0.01	**0.17**	**11.6**	16	<0.004	0.01	0.03	**0.35**	7
Araria	2	<0.01	<0.01	<0.01	<0.01	0	<0.004	<0.004	<0.004	<0.004	0
Arwal	2	<0.01	<0.01	<0.01	<0.01	0	<0.004	<0.004	<0.004	0.01	0
Aurangabad	8	<0.01	<0.01	<0.01	<0.01	0	<0.004	0.01	0.02	0.08	0
Banka	6	<0.01	<0.01	<0.01	0.03	0	<0.004	0.01	0.01	0.02	0
Begusarai	4	0.12	**0.16**	**0.16**	**0.21**	75	<0.004	<0.004	<0.004	<0.004	0
Bhagalpur	6	<0.01	<0.01	<0.01	0.04	0	0.01	0.07	0.07	**0.15**	17
Bhojpur	3	<0.01	<0.01	<0.01	<0.01	0	0.01	0.01	0.01	0.01	0
Buxar	15	<0.01	**0.41**	**0.51**	**1.79**	80	<0.004	<0.004	0.004	0.04	0
Darbhanga	3	0.06	0.07	0.06	0.07	0	<0.004	<0.004	<0.004	<0.004	0
East Champaran	12	<0.01	0.04	0.05	**0.27**	8	<0.004	0.01	0.01	0.08	0
Gaya	4	<0.01	<0.01	<0.01	<0.01	0	0.02	0.03	0.03	0.04	0
Gopalganj	7	<0.01	0.02	0.02	0.07	0	<0.004	0.02	0.04	**0.19**	14
Jamui	6	<0.01	<0.01	<0.01	<0.01	0	<0.004	<0.004	0.01	0.04	0
Jehanabad	2	<0.01	<0.01	<0.01	<0.01	0	0.01	0.05	0.05	0.09	0
Kaimur	2	<0.01	<0.01	<0.01	<0.01	0	0.01	0.02	0.02	0.03	0
Katihar	5	<0.01	<0.01	**0.55**	**2.72**	20	<0.004	<0.004	0.04	**0.22**	20
Khagaria	2	0.09	0.12	0.12	**0.14**	50	<0.004	<0.004	<0.004	<0.004	0
Kishanganj	3	<0.01	<0.01	<0.01	<0.01	0	<0.004	<0.004	<0.004	<0.004	0
Lakhisarai	2	<0.01	<0.01	<0.01	<0.01	0	0.03	0.03	0.03	0.03	0
Madhepura	1	--	<0.01	<0.01	--	0	--	0.004	0.004	--	0
Madhubani	6	0.06	0.11	**0.15**	**0.39**	50	<0.004	<0.004	<0.004	<0.004	0
Munger	4	<0.01	<0.01	<0.01	<0.01	0	0.03	0.03	0.06	**0.14**	25
Muzaffarpur	15	<0.01	<0.01	0.02	0.10	0	<0.004	0.04	0.05	**0.28**	13
Nalanda	5	<0.01	<0.01	<0.01	<0.01	0	0.04	0.06	**0.14**	**0.32**	40
Nawada	5	<0.01	<0.01	<0.01	<0.01	0	0.01	**0.15**	**0.13**	**0.21**	80
Patna	62	<0.01	<0.01	0.05	**1.22**	5	<0.004	0.01	0.02	**0.15**	2
Purnia	4	<0.01	0.01	0.03	0.08	0	<0.004	0.004	0.01	0.03	0
Rohtas	7	<0.01	<0.01	<0.01	<0.01	0	<0.004	0.02	0.01	0.03	0
Saharsa	2	<0.01	0.01	0.01	0.03	0	<0.004	<0.004	<0.004	0.005	0
Samastipur ^1^	6	<0.01	0.05	0.07	**0.18**	33	<0.004	0.01	0.02	0.06	0
Samastipur ^2^	10	<0.01	**0.16**	**2.23**	**11.6**	60	--	--	--	--	--
Saran	14	<0.01	0.05	**0.23**	**2.50**	21	<0.004	<0.004	0.02	**0.18**	7
Sheikpura	1	--	<0.01	<0.01	--	0	--	0.04	0.04	--	0
Sheohar	2	0.04	0.07	0.07	0.10	0	<0.004	<0.004	<0.004	0.004	0
Sitamarhi	8	0.03	**0.15**	**0.22**	**0.82**	50	<0.004	<0.004	<0.004	0.01	0
Siwan	7	<0.01	<0.01	0.03	**0.13**	14	0.01	0.02	0.05	**0.16**	14
Supaul	6	<0.01	0.01	0.10	**0.44**	33	<0.004	0.02	0.07	**0.35**	17
Vaishali	10	<0.01	<0.01	0.08	**0.82**	10	<0.004	0.09	0.10	**0.23**	40
West Champaran	10	<0.01	0.04	0.10	**0.40**	30	<0.004	0.01	0.01	0.02	0

^1^ Samastipur random sampling set only; ^2^ Samastipur set including four samples from a known As “hot spot”.
